# The Leydig cell biomarker INSL3 as a predictor of age-related morbidity: Findings from the EMAS cohort

**DOI:** 10.3389/fendo.2022.1016107

**Published:** 2022-11-08

**Authors:** Richard Ivell, Kee Heng, Katie Severn, Leen Antonio, Gyorgy Bartfai, Felipe F. Casanueva, Ilpo T. Huhtaniemi, Aleksander Giwercman, Mario Maggi, Daryl B. O’Connor, Terence W. O’Neill, Margus Punab, Giulia Rastrelli, Jolanta Slowikowska-Hilczer, Jos Tournoy, Dirk Vanderschueren, Frederick C. W. Wu, Ravinder Anand-Ivell

**Affiliations:** ^1^ School of Biosciences, University of Nottingham, Sutton Bonington, United Kingdom; ^2^ School of Mathematics, University of Nottingham, Nottingham, United Kingdom; ^3^ Department of Chronic Diseases and Metabolism, Laboratory of Clinical and Experimental Endocrinology, KU Leuven, Leuven, Belgium; ^4^ Department of Endocrinology, University Hospitals Leuven, Leuven, Belgium; ^5^ Department of Obstetrics, Gynaecology and Andrology, Albert Szent-Gyorgy Medical University, Szeged, Hungary; ^6^ Department of Medicine, Santiago de Compostela University, Complejo Hospitalario Universitario de Santiago (CHUS); CIBER de Fisiopatología Obesidad y Nutricion (CB06/03), Instituto Salud Carlos III, Santiago de Compostela, Spain; ^7^ Institute of Reproductive and Developmental Biology, Department of Metabolism, Digestion and Reproduction, Imperial College London, London, United Kingdom; ^8^ Department of Translational Medicine, Lund University, Malmö, Sweden; ^9^ Andrology Unit, “Mario Serio” Department of Experimental and Clinical Biomedical Sciences, University of Florence, Florence, Italy; ^10^ School of Psychology, University of Leeds, Leeds, United Kingdom; ^11^ Centre for Epidemiology Versus Arthritis, The University of Manchester & NIHR Manchester Biomedical Research Centre, Manchester University NHS Foundation Trust, Manchester, United Kingdom; ^12^ Andrology Clinic, Tartu University Hospital; and Institute of Clinical Medicine, and Institute of Biomedicine and Translational Medicine, University of Tartu, Tartu, Estonia; ^13^ Department of Andrology and Reproductive Endocrinology, Medical University of Łódź, Łódź, Poland; ^14^ Department of Geriatrics, University Hospitals Leuven, and Department of Public Health and Primary Care, KU Leuven, Leuven, Belgium; ^15^ Department of Endocrinology, Manchester University NHS Foundation Trust, Manchester, United Kingdom

**Keywords:** sexual function, hypertension, hypogonadism, cardiovascular disease, frailty, bone mineral density

## Abstract

**Background:**

Insulin-like peptide 3 (INSL3) is a constitutive hormone secreted in men by the mature Leydig cells of the testes. It is an accurate biomarker for Leydig cell functional capacity, reflecting their total cell number and differentiation status.

**Objectives:**

To determine the ability of INSL3 to predict hypogonadism and age-related morbidity using the EMAS cohort of older community-dwelling men.

**Materials & methods:**

Circulating INSL3 was assessed in the EMAS cohort and its cross-sectional and longitudinal relationships to hypogonadism, here defined by testosterone (T) <10.5nmol/l, and a range of age-related morbidities determined by correlation and regression analysis.

**Results & discussion:**

While INSL3 is an accurate measure of primary hypogonadism, secondary and compensated hypogonadism also indicate reduced levels of INSL3, implying that testicular hypogonadism does not improve even when LH levels are increased, and that ageing-related hypogonadism may combine both primary and secondary features. Unadjusted, serum INSL3, like calculated free testosterone (cFT), LH, or the T/LH ratio reflects hypogonadal status and is associated with reduced sexual function, bone mineral density, and physical activity, as well as increased occurrence of hypertension, cardiovascular disease, cancer, and diabetes. Using multiple regression analysis to adjust for a range of hormonal, anthropometric, and lifestyle factors, this relationship is lost for all morbidities, except for reduced bone mineral density, implying that INSL3 and/or its specific receptor, RXFP2, may be causally involved in promoting healthy bone metabolism. Elevated INSL3 also associates with hypertension and cardiovascular disease. When unadjusted, INSL3 in phase 1 of the EMAS study was assessed for its association with morbidity in phase 2 (mean 4.3 years later); INSL3 significantly predicts 7 out of 9 morbidity categories, behaving as well as cFT in this regard. In contrast, total T was predictive in only 3 of the 9 categories.

**Conclusion:**

Together with its low within-individual variance, these findings suggest that assessing INSL3 in men could offer important insight into the later development of disease in the elderly.

## Introduction

Insulin-like peptide 3 (INSL3) is a peptide hormone produced in men uniquely by the steroidogenic Leydig cells of the testes (1). Unlike testosterone (T), however, it is secreted into the bloodstream in a constitutive manner which is acutely independent of the pituitary gonadotrophins that are partly responsible for the high within-individual, biological variance observed with the steroid hormone. INSL3 production only reflects the absolute number of Leydig cells and their mature differentiation status (2). Leydig cell proliferation and differentiation from relatively undifferentiated resident stem cells occur during childhood and adolescence in response to the chronic effects of LH to promote puberty and subsequent sexual maturity. Consequently, circulating INSL3 accurately reflects the progression of puberty, acting much like a biochemical Tanner staging, reaching a maximum in young adulthood around 18-20 years of age (3, 4). Since there appears to be no further increase in Leydig cell number or their differentiation status thereafter throughout adult life - rather there appears to be a reduction in mature Leydig cells in later age – there is no further increase in circulating INSL3. And from 30-40 years onwards, INSL3 concentration in the blood appears to decline at approximately 15% per decade, matching the apparent loss of mature Leydig cell numbers (5, 6). This is a larger decline than that observed for T, since age- dependent T reduction is continually compensated by elevated gonadotropins due to reduced testicular feedback to the hypothalamus and pituitary. Consequently, the concomitant decrease of circulating INSL3 concentration can be seen as an accurate reflection of the reduced number and differentiation status of the Leydig cell population and hence also of their functional capacity to produce T (7, 8). In this regard, INSL3 concentration can be seen as somewhat like the T/LH ratio (9). However, it has the great advantage that being constitutive and having low within-individual variation, it does not suffer the high biological variance accruing to T or its derivative parameters.

The EMAS (European Male Aging Study) cohort of community-dwelling men comprises more than 3000 men, aged 40-79 years at the time of recruitment, from 8 centers in east, west, north and south Europe (10). Men were recruited in 2003-2004 and again 4-5 years later for a second phase of the study. In both phases blood was collected for hormonal measurements, and subjects were assessed for anthropometric parameters and asked to complete questionnaires relating to their health, lifestyle, and diet. In a first analysis, after adjustment for age and center, INSL3 was shown to be correlated positively with T, calculated free T (cFT), and the T/LH ratio, but negatively with LH, FSH, BMI or waist circumference, and smoking (6). When specifically looking for predictive parameters using multiple regression analysis, these relationships persisted, except that now FSH became a positive rather than negative predictor. These results reveal that unlike T, which is acutely being driven by pituitary LH output, INSL3 is independent of such acute control but rather reflects the overall long-term status of the hypothalamus-pituitary-gonadal (HPG) axis as men age. For example, in younger healthy men, when T is high and LH relatively low, then INSL3 is also high, chronically reflecting this healthy status. But in older age, when T levels are maintained within the normal range only by elevated LH and FSH, INSL3 is significantly reduced.

In summary, therefore, circulating INSL3 represents a stable parameter which is reflecting the chronic functional status of the Leydig cells within the testes, and hence their capacity to produce T. Its low biological and technical variance, and hence within-individual consistency over time also means that it could predict better than T future Leydig cell functional status. It is known that low T is associated with a range of age-associated morbidities, such as diabetes, obesity, cognitive decline, and cardiovascular disease, besides sexual function (11, 12). Since INSL3, because of its Leydig cell origin, correlates with T at a population level (4–6), we have used data from the EMAS cohort to determine whether INSL3, being a dissimilar parameter of Leydig cell function, could provide novel and improved information about the correlation of testicular function with age-related disease.

## Materials and methods

### Subjects, anthropometric data

The EMAS cohort has been described in detail elsewhere (10). In 2003-2005, the study recruited 3369 community-dwelling men aged between 40 and 79 years from 8 European centers across Europe (10). Postal and interviewer-assisted questionnaires were used to assess disease occurrence, lifestyle factors, as well as physical and cognitive performance (10). Blood samples were also collected and used for measurement of hormones and other clinical parameters. In a second phase of the study, men were recalled in 2008-2010, on average 4.3 years (range 3.0-5.7 years) after the first phase, and similar parameters measured again (13). Subject retention in the second phase was 81.2%. In the present study, 1252 serum samples (37.2%) from 4 centers of the first phase were available for INSL3 measurement, together with 2283 serum samples (83.4%) from the second phase from all 8 centers (250-300 per centre). Consequently, emphasis is given here to the EMAS second phase for which most cross-sectional subject information is available; samples from the first phase are used for longitudinal comparison. Descriptive statistics for the second phase parameters have been presented previously (6) and are reproduced here together with relevant parameters for the available samples for phase 1 as **Supplementary Table S1**. These are essentially similar to those from previous studies (e.g. 13). Body weight was measured to the nearest 0.1 kg, and height to the nearest 1 mm. BMI (kg/m^2^) was calculated from body weight and height. Waist circumference (WC; cm) is given as the mean of triplicate measurements for each subject.

### Questionnaires to evaluate smoking, alcohol, etc.

Postal questionnaires were used to determine whether the subject was a current, past or non-smoker. Here, for statistical analysis (nominal categories in brackets), current smokers (1) were compared with pooled past smokers and non-smokers (0). Similarly, alcohol intake was assessed in terms of weekly consumption; for multivariate analysis, 0-1 drinks per week was regarded as ‘low’ (0), 2-4 drinks per week as ‘moderate’ (1), and >4 drinks per week as ‘high’ (2).

### Hormone assays

As detailed previously (10, 13), phlebotomy was carried out prior to 10.00am and sera stored at -80°C. Total testosterone (T) was quantified by GC-MS (14) for all samples in one laboratory (within- and between-run COV, was < 3.5%; LOD, 0.17 nmol/l). LH, FSH and SHBG were measured by Modular E170 platform (Roche Diagnostics, Mannheim, Germany) electrochemiluminescence immunoassay (within- and between-assay COV: LH, 1.88% and 3.01% (LOD, 0.10 U/l); FSH, 0.9% and 1.9% (LOD 0.10 U/l); SHBG, 1.70% and 3.18% (LOD 0.35 nmol/l), respectively) (12). Calculated free testosterone (cFT) was derived from total testosterone (T), SHBG, and albumin, using the Vermeulen formula (15). INSL3 was assessed by specific time-resolved fluorescent immunoassay (1) (inter- and intra-plate COV, <8% and <3%, respectively, LOD, 20pg/ml). This well-established and validated assay yields essentially identical values to a new LC-MS/MS procedure (16).

Subjects were defined as eugonadal (T>10.5 nmol/l; LH<9.4U/l), primary hypogonadal (T<10.5 nmol/l; LH>9.4 nmol/l), secondary hypogonadal (T<10.5 nmol/l; LH<9.4 nmol/l), and compensated hypogonadal (T>10.5 nmol/l; LH>9.4 nmol/l) according to the criteria of Tajar et al. (17).

### Frailty and bone assays

Frailty was estimated by assessing physical mobility using the PASE (Physical Activity Scale for the Elderly) index (18) with an interviewer-guided questionnaire. For binomial logistic regression, the continuous PASE variable was classified as 0 (PASE <78; reduced physical activity) or 1 (PASE >78) (19). Bone mineral density was assessed by ultrasound examination of the left calcaneal (heel) bone using a Sahara clinical sonometer (10). The same model was used by each center and calibrated daily using the phantom provided by the manufacturer. Parameters measured were the speed of sound (SOS) and broadband ultrasound attenuation (BUA). From these two parameters also an estimate of bone mineral density (BMD; g/cm^2^) was derived using the formula BMD = 0.002592×(BUA+SOS)−3.687 (20). *In vivo* COVs were 0.3%, 2.8%, and 3.4% for SOS, BUA, and BMD, respectively. Subsequently, for use in the binomial logistic regression of various morbidities, the BMD data only were categorized into lower (1) and upper 50 percentiles (0), representing lower and higher bone mineral density, respectively.

### Sexual function

Each subject received a personal sexual function questionnaire (SFQ) specifically developed and validated for EMAS (21). The SFQ included not only questions related to sexual function or dysfunction, for example, regarding erectile or ejaculatory issues, frequency of sexual behaviour, libido, sexual satisfaction, and masturbation, but also regarding sexual distress or recent change in sexual function. Principal component analysis of the findings from the initial validation study using this SFQ in the EMAS cohort revealed that the resulting data could be summarized into four discrete categories: *overall sexual function* (*osf*), *sexual function distress* (*sfd*), *change in sexual function* (*csf*), and *masturbation frequency* (*m*) (21). These statistical categories were used here as nominal variables. Subsequently, for use in the binomial logistic regression of various morbidities, the *osf* data only were categorized into lower (1) and upper 50 percentiles (0).

### Assessment of morbidity

Self-reported morbidity was obtained by postal questionnaire and based on previous medical diagnosis (13). This included ever having had cancer, diabetes, high blood pressure, and cardiovascular disease (CVD). These are reported as simple yes/no binary variables. Depression was estimated by interviewer-assisted questionnaire using the Beck Depression Inventory (BDI-II) (22). For binary logistic regression, men were identified as having ‘no depression’ (score 0–13) or ‘mild to severe depression’ (score 14–63) (22, 23). A separate category was also prepared for subjects having two or more comorbidities; in addition to the above listed conditions, this included respiratory ailments such as asthma or bronchitis, liver, kidney or prostate disease, and thyroid disorders. Ever having had stroke was included within cardiovascular disease.

### Statistics

Descriptive statistics for the analyzed subjects from phase 2 were shown previously (6) and reproduced here as **Supplementary Table S1**. The hormone parameters INSL3, T, cFT, SHBG, LH, FSH, and the T/LH ratio, being significantly skewed, were transformed to their natural logarithm for regression and correlation analysis. We had previously shown that most (73.2%) of the variance from significant center effects for INSL3 could be predicted from BMI, smoking, and alcohol consumption (6). Consequently, center effects were excluded from regression analyses as collinear with these parameters, with the small residual variance being considered a random influence. To check this, where appropriate, analyses were repeated with and without center included with no difference in the results. For the comparison of hypogonadal and eugonadal men, since the former were significantly older, INSL3 data were also normalized to a standard individual of 65 years by interpolation from the center-specific regressions of INSL3 against age. Morbidity parameters were first compared by simple correlation analysis, followed by partial correlation, adjusting for age. For continuous variables, multiple regression analysis was used, with T, but not cFT, nor the T/LH ratio, and BMI, but not waist circumference (WC), included since these parameters are collinear. In the cross-sectional analysis, unadjusted Odds Ratios (OR) were calculated from the 95% confidence intervals of the beta-coefficients generated from binomial logistic regression of INSL3, T, cFT, LH, and the T/LH ratio against the binomial categorical morbidity variables. For subsequent modelling of morbidities, total variance accounted for in the models was estimated by the Nagelkerke quasi-R^2^ values. Finally, when assessing phase 2 morbidity OR from phase 1 hormone parameters, the latter were expressed as quartiles, comparing the lowest quartile against the remainder as binary categories for INSL3, T, cFT and the T/LH ratio, and the upper quartile for LH. All statistics were carried out using either the SPSS package (version 27; IBM SPSS Statistics for Windows; IBM Corporation, Armonk, NY) or GraphPad Prism (version 8.2; GraphPad Software, La Jolla, CA).

## Results

### INSL3 and hypogonadism

**Figure 1:**
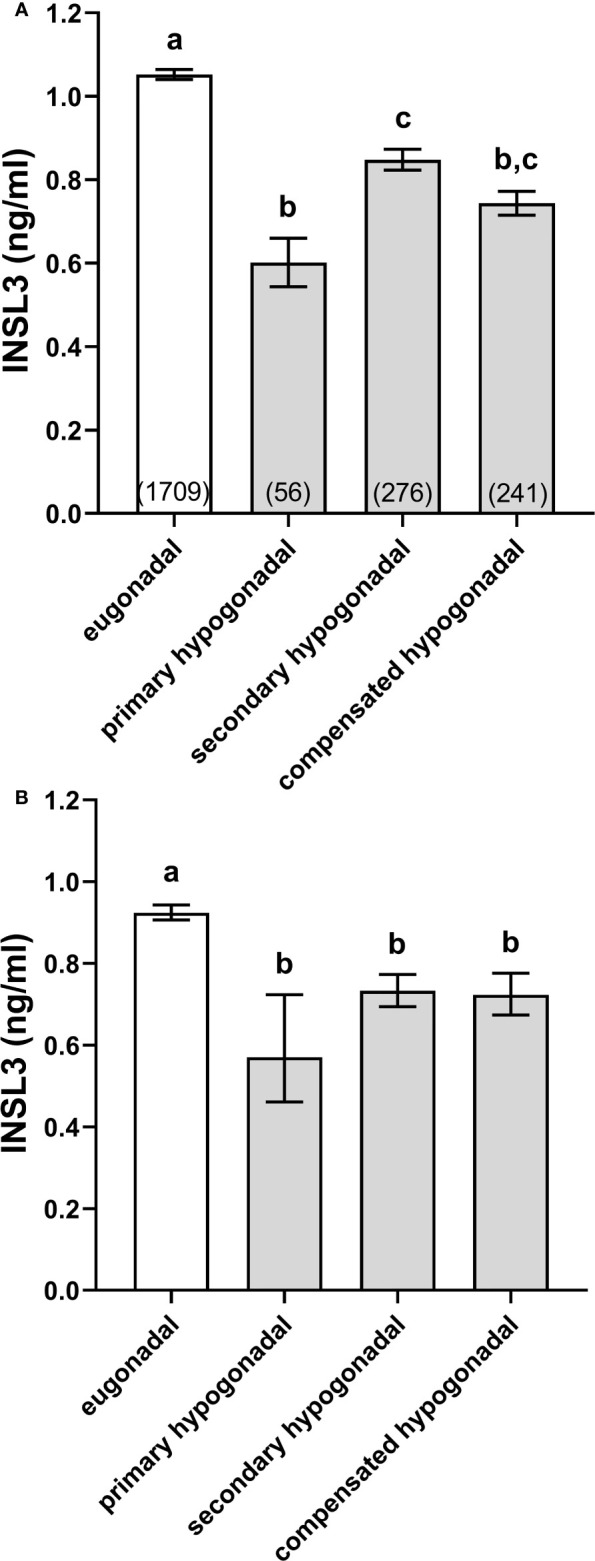
Simple means ± SEM for INSL3 **(A)** and age-adjusted INSL3 means and 95% confidence intervals **(B)** calculated by interpolation from individual INSL3 x age regressions for age 65 years, representing eugonadal (T>10.5 nmol/l; LH ≤ 9.4U/l), primary hypogonadal (T ≤ 10.5 nmol/l; LH>9.4 nmol/l), secondary hypogonadal (T ≤ 10.5 nmol/l; LH ≤ 9.4 nmol/l), and compensated hypogonadal (T>10.5 nmol/l; LH>9.4 nmol/l) subjects according to the criteria of Tajar et al. (17). The numbers of subjects in each category are indicated in parentheses. (a-c) different letters above the columns indicate significant differences between the columns, p<0.001; similar letters indicate no significant difference.

The EMAS cohort was separated into eugonadal (high T, low/normal LH), primary hypogonadal (low T, high LH), secondary hypogonadal (low T, low/normal LH), and compensated hypogonadal subjects (high T, high LH), following the precise criteria of Tajar et al. (17) (see Methods and Materials). All three hypogonadal categories had significantly reduced INSL3 concentrations compared to the eugonadal subjects (**Figure 1**), though these were mostly not different from one another, except that primary hypogonadism appeared to be reduced compared to secondary hypogonadism (**Figure 1A**). Since primary hypogonadism is more common amongst older men, the results were re-analyzed, correcting for age, by plotting the interpolated values from INSL3 against age regressions within each category, normalized to an individual of 65 years (**Figure 1B**). The results showed that there were now no significant differences between all hypogonadal categories.

### Physical activity and bone health

Multiple regression analysis was carried out to determine which, if any, of the available parameters significantly contributed to the variance in physical activity, as measured by the PASE index, here as a continuous variable with a high value indicating increased mobility, or in bone mineral density of the calcaneal (heel) bone, assessed by ultrasound (**Table 1**). For physical activity, only age, smoking, and BMI were contributory factors, all negatively. For mean bone mineral density (BMD) age, smoking, and SHBG contributed negatively, and BMI, INSL3, T, and alcohol contributed positively, in that order. For the speed of sound (SOS) ultrasound parameter, only age was significantly involved and that negatively. Finally, for bone ultrasound attenuation (BUA), smoking, FSH, and SHBG all contributed negatively, whereas BMI and INSL3 both contributed positively (**Table 1**). Importantly, whereas T positively contributed to the variance only for BMD, INSL3 contributed positively to both BMD and BUA independently of other parameters. When cFT is substituted for T and SHBG (because of collinearity) in this analysis, results are essentially similar (**Supplementary Table S2**) the only difference being that now cFT replaces SHBG, rather than T, as a significant parameter in regard to BUA.

**Table 1:** Multiple regression analysis modelling the PASE (Physical Activity Scale for the Elderly) index, as well as BMD (bone mineral density), SOS (ultrasound speed of sound), and BUA (bone ultrasound attenuation) for heel bone ultrasound assessment as continuous dependent variables, using hormonal, anthropometric, and lifestyle parameters as independent variables, as indicated, including INSL3.

	PASE (10.6%)			BMD (4.0%)			sos (0.2%)			BUA (4.6%)		
	std. β	t	*p-*value	std.β	t	*p-*value	std.	t	*p-*value	std. β	t	*p-*value
**age**	-0.326	-14.14	< 0.001	-0.067	-2.44	0.015	-0.049	-2.12	0.034	excl.	excl.	excl.
**INSL3**	excl.	excl.	excl.	0.066	2.48	0.013	excl.	excl.	excl.	0.083	3.44	< 0.001
**T**	excl.	excl.	excl.	0.060	2.00	0.046	excl.	excl.	excl.	excl.	excl.	excl.
**SHBG**	excl.	excl.	excl.	-0.086	-2.75	0.006	excl.	excl.	excl.	-0.060	-2.39	0.017
**LH**	excl.	excl.	excl.	excl.	excl.	excl.	excl.	excl.	excl.	excl.	excl.	excl.
**FSH**	excl.	excl.	excl.	excl.	excl.	excl.	excl.	excl.	excl.	-0.071	-2.90	0.004
**smoking**	-0.100	4.29	< 0.001	-0.053	2.23	0.026	excl.	excl.	excl.	-0.081	-3.51	< 0.001
**alcohol**	excl.	excl.	excl.	0.046	2.01	0.045	excl.	excl.	excl.	excl.	excl.	excl.
**BMI**	-0.072	-3.16	0.002	0.103	4.18	< 0.001	excl.	excl.	excl.	0.116	4.74	< 0.001

std.b is the standardized beta coefficient for the model with its t value and significance (*p* value). excl. indicates a parameter excluded in the final optimal model. The values in yellow shading denote statistical significance.

### INSL3 and sexual function

A previous study (21) had shown that of the many factors identified by questionnaire that could be linked to sexual function, principal component analysis was able to group these into four discrete categories: *osf* (*overall sexual functioning*), *csf* (*change in sexual functioning*), *sfd* (*sexual functioning distress*), and *m* (*frequency of masturbation*). Simple unadjusted correlation analysis (**Table 2A**) showed that subject age, T, cFT, INSL3, SHBG, LH, FSH, the T/LH ratio, BMI and alcohol intake were all associated with *osf*, *csf* (except for T), and *m* (except for BMI), though only the T/LH ratio, BMI, and also smoking correlated with the remaining sexual parameter, *sfd* (*sexual function distress*). However, correction for age (**Table 2B**) removed the significant association for INSL3 with *csf*, and left significant associations for *osf* with BMI, T, cFT, LH, FSH and the T/LH ratio, as well as with smoking and alcohol consumption. There were now significant correlations for *sfd* only with BMI and smoking, none at all for *csf*, and for *m* only with INSL3, SHBG, and alcohol consumption (**Table 2B**). When multiple regression analysis (**Table 2C**) was used to analyze the contributions to the variances of the four sexual parameters, now including age, INSL3, T, SHBG, LH, FSH, BMI, smoking, and alcohol intake as independent and potential contributory variables, INSL3, BMI and FSH indicated no influence on *osf* (**Table 2C**). Age, T, SHBG, LH, smoking, and alcohol intake together supported 32.4% of the variance in *osf*, estimated from the Pearson R^2^. For *sfd* (explained variance 1.7%), only BMI and smoking contributed; for *csf* (explained variance 6.8%) only age and alcohol intake, and for *m* (explained variance 0.8%) only age, INSL3 and alcohol intake appeared to contribute.

**Table 2:** (A) Unadjusted simple correlation analysis for the principal component parameters *osf* (overall sexual function), *sfd* (sexual function distress), *csf* (change in sexual function) and *m* (frequency of masturbation) against hormonal, anthropometric, and lifestyle parameters, as indicated (for details, see Materials and Methods).

A. Correlation analysis (unadjusted)													
	*osf*			*sfd*			*csf*				*m*		
	R	*p* value	N	R	*p* value	N	R	*p* value		N	R	*p* value	N
age	-0.538	< 0.001	1622	0.037	0.111	1874	0.261	< 0.001		1901	-0.272	< 0.001	2083
INSL3	0.231	< 0.001	1622	0.013	0.560	1874	-0.091	< 0.001		1901	0.168	< 0.001	2083
BMI	-0.055	0.028	1599	0.112	< 0.001	1847	0.047	0.044		1871	-0.009	0.682	2050
T	0.119	< 0.001	1622	-0.027	0.244	1846	-0.035	0.127		1901	0.053	0.016	2083
cFT	0.271	< 0.001	1602	-0.030	0.196	1846	-0.124	< 0.001		1872	0.153	< 0.001	2050
SHBG	-0.202	< 0.001	1602	-0.006	0.785	1846	0.112	< 0.001		1872	-0.143	< 0.001	2050
LH	-0.190	< 0.001	1602	0.032	0.165	1846	0.080	< 0.001		1872	-0.087	< 0.001	2050
FSH	-0.263	< 0.001	1600	0.022	0.356	1845	0.102	< 0.001		1870	-0.120	< 0.001	2048
T/LH ratio	0.252	< 0.001	1602	-0.046	0.049	1846	-0.091	< 0.001		1872	0.113	< 0.001	2050
smoking	0.009	0.712	1582	0.067	0.004	1828	-0.002	0.947		1851	0.005	0.809	2030
alcohol	0.121	< 0.001	1457	0.017	0.487	1656	-0.065	0.008		1680	0.110	< 0.001	1844
**B. Partial correlation analysis (adjusted for age)**													
	*osf*			*sfd*			*csf*				*m*		
	**R**	***p* value**	**df**	**R**	***p* value**	**df**	**R**	***p* value**		**df**	**R**	***p* value**	**df**
INSL3	0.067	0.018	1253	0.022	0.434	1253	0.018	0.521		1253	0.069	0.015	1253
BMI	-0.066	0.019	1253	0.120	< 0.001	1253	0.046	0.104		1253	-0.001	0.982	1253
T	0.091	< 0.001	1253	-0.042	0.132	1253	-0.034	0.227		1253	0.002	0.935	1253
cFT	0.119	< 0.001	1253	-0.033	0.248	1253	-0.040	0.153		1253	0.042	0.133	1253
SHBG	-0.003	0.905	1253	-0.035	0.215	1253	-0.021	0.463		1253	-0.060	0.032	1253
LH	-0.058	0.038	1253	0.030	0.293	1253	-0.009	0.744		1253	-0.009	0.763	1253
FSH	-0.072	0.011	1253	0.004	0.885	1253	-0.036	0.199		1253	-0.026	0.383	1253
T/LH ratio	0.114	< 0.001	1253	-0.055	0.051	1253	-0.015	0.588		1253	0.009	0.750	1253
smoking	-0.089	0.002	1253	0.066	0.019	1253	0.042	0.136		1253	-0.051	0.074	1253
alcohol	0.105	< 0.001	1253	0.001	0.976	1253	-0.034	0.223		1253	0.116	< 0.001	1253
**C. Multiple regression analysis**													
		***osf*** **(32.4%)**			***sfd*** **(1.7%)**			***csf*** **(6.8%)**				***m*** **(0.8%)**	
	**Std β**	**t**	***p* value**	**Std β**	**t**	***p* value**	**Std β**	**t**	**pvalue**		**Std β**	**t**	**pvalue**
age	-0.508	-19.12	< 0.001	excl	excl	excl	0.257	10.71	< 0.001		-0.233	-9.56	< 0.001
INSL3	excl	excl	excl	excl	excl	excl	excl	excl	excl		0.070	2.88	0.004
BMI	excl	excl	excl	0.122	4.86	< 0.001	excl	excl	excl		excl	excl	excl
T	0.137	4.83	< 0.001	excl	excl	excl	excl	excl	excl		excl	excl	excl
SHBG	-0.063	-2.08	0.038	excl	excl	excl	excl	excl	excl		excl	excl	excl
LH	-0.055	-2.31	0.021	excl	excl	excl	excl	excl	excl		excl	excl	excl
FSH	excl	excl	excl	excl	excl	excl	excl	excl	excl		excl	excl	excl
smoking	-0.076	-3.35	< 0.001	0.075	2.98	0.003	excl	excl	excl		excl	excl	excl
alcohol	0.097	4.41	< 0.001	excl	excl	excl	-0.055	-2.27	0.023		0.100	4.37	< 0.001

Significant associations are highlighted in yellow with larger font. R, Pearson’s correlation coefficient; p value, statistical significance, N, number of subjects included. **(B)** Partial correlation analysis (as above, adjusted for age). R, Pearson’s correlation coefficient; p value, statistical significance, df, degrees of freedom. **(C)** Multiple regression analysis to model the relationships between the same dependent and independent variables, as above. The percentages in parentheses indicate the proportion of variance explained by the models based on the Pearson R^2^. Std.b is the standardized beta coefficient for the model with its t value and significance (*p* value). excl. indicates a parameter excluded in the final optimal model.

### INSL3 and morbidity

**Figure 2:**
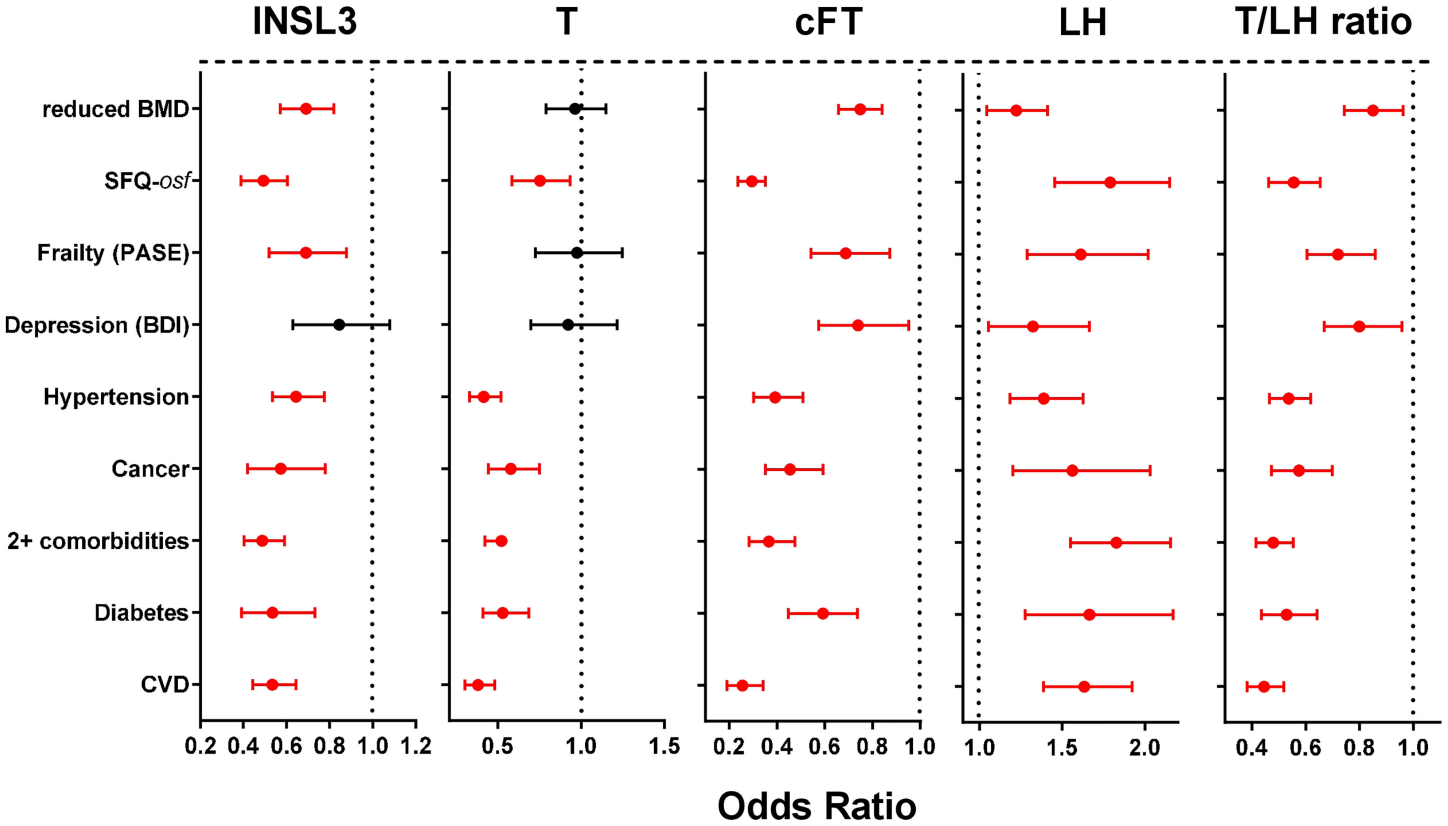
Odds ratios (OR; with 95% confidence intervals) calculated from simple binomial logistic regressions of the indicated binomial categorical variables representing different phase 2 morbidities (present vs absent), as indicated on the Y axis, against the various phase 2 predictors: INSL3, T, cFT, LH, or the T/LH ratio as continuous variables. Significant OR are indicated in red. Bone mineral density (BMD) is here expressed as a binomial categorical variable with the lower 50^th^ percentile represented as 1, and the higher 50^th^ percentile represented as 0. Similarly, overall sexual function (SFQ-*osf*) is also expressed as a binomial categorical variable with the lower 50th percentile represented as 1, and the higher 50th percentile represented as 0.

Univariate binomial logistic regression was first used to calculate unadjusted Odds Ratios (OR) for the association of various hormone parameters linked to the HPG axis, as well as INSL3 (all as continuous variables) with a range of self-reported morbidities and related parameters (**Figure 2**) all expressed as binomial categorical variables. These OR reflect the association of the parameter with the occurrence of a particular condition. Whereas cFT, LH and the T/LH ratio indicated significant OR for all listed categories of morbidity, INSL3 showed no significant relationship only in regard to depression. In contrast, T indicated a significant OR for overall sexual function (*osf*), hypertension, ever having had cancer, diabetes, cardiovascular disease, and 2 or more morbidities, but not for reduced bone mineral density (BMD), frailty, nor depression.

**Figure 3:**
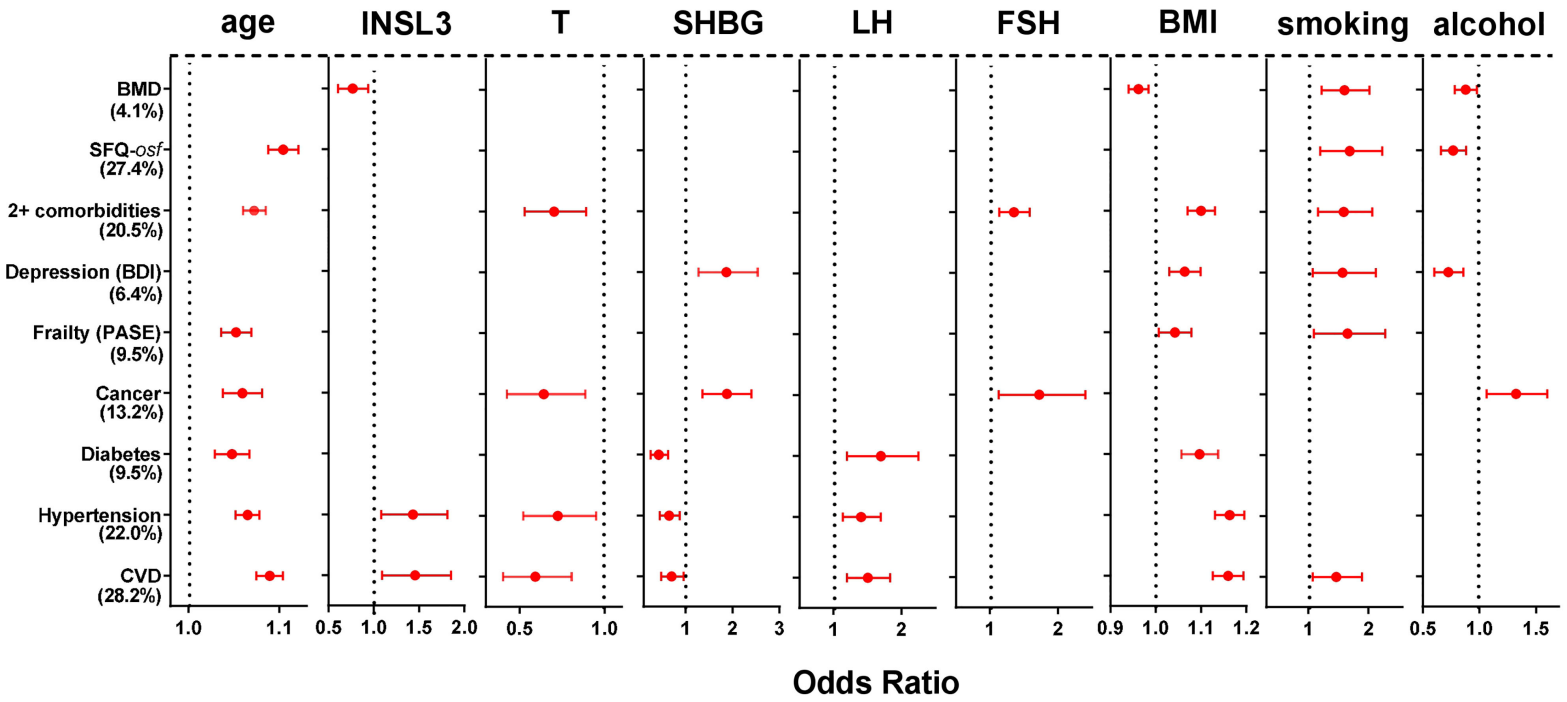
Significant Odds Ratios (OR; mean and 95% confidence intervals) calculated from multivariate binomial logistic regressions of the indicated binomial categorical variables from phase 2 representing different morbidities, as indicated on the Y axis, against various hormonal, anthropometric and lifestyle parameters as independent continuous predictors, as indicated, also from phase 2. Significant OR are indicated in red; non-significant relationships are excluded (not shown). The estimated variance (percentage, based on the Nagelkerke pseudo-R^2^) accounted for by the models is indicated in parentheses. BMD and SFQ-*osf* are expressed as binomial categorical variables, as in the legend to **Figure 2**.

The various categories of morbidity were further analyzed using multivariate binomial logistic regression (**Figure 3**) to determine which hormonal and/or other factors of those assessed are most likely contributing to the cohort variance and thus most likely to be involved in a causal relationship. cFT, the T/LH ratio, and waist circumference were excluded as being collinear with T, LH and/or BMI. Of the parameters included, both elevated age and BMI significantly contributed to 7 out of the 9 morbidity categories, smoking to 6, SHBG to 5, and T and alcohol intake to 4. Both T, smoking, as well as both gonadotropins contributed in a manner consistent with the generally negative effect of hypogonadism or smoking on health. Whereas increasing SHBG was associated with an increased risk of depression or ever having had cancer, increasing SHBG was associated also with a reduced risk of diabetes, hypertension, and cardiovascular disease. Alcohol intake (4 days per week or more vs. < 4 days per week) was mostly not associated with the listed types of morbidity, except for ever having had cancer, depression, overall sexual function, and reduction in bone mineral density. Of particular interest is the observation that increasing INSL3 is mostly not associated specifically with morbidity once age, T, BMI and/or smoking are included in the equations. The exceptions are an increased risk of hypertension and cardiovascular disease, both of whose regression equations account for >20% of the cohort variance, and which already include contributions from T and SHBG (negatively), and age, LH, and BMI (positively). Here increased INSL3 appears to contribute positively towards both morbidities, though to no other, either positively or negatively. In contrast, INSL3 contributes negatively to a loss of bone mineral density, i.e. it is associated with higher BMD.

**Figure 4:**
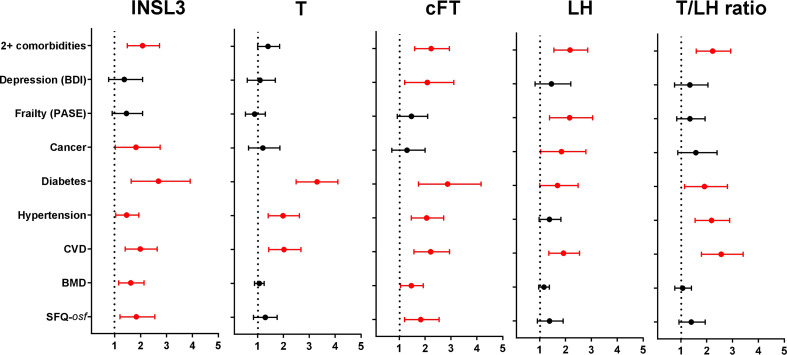
Odds Ratios (OR; mean and 95% confidence intervals) calculated from multivariate binomial logistic regressions of the indicated binomial categorical variables representing different morbidities from phase 2, as indicated on the Y axis, against various hormonal parameters from phase 1 as independent predictors, here represented as binomial categorical variables. INSL3, T, cFT, and T/LH ratio: lowest quartiles vs remainder; LH: highest quartile vs remainder. Significant OR are indicated in red; non-significant relationships are excluded (not shown). BMD and SFQ-*osf* are expressed as binomial categorical variables, as in the legend to **Figure 2**.

Finally, although there was only a relatively short period (average 4.3 years) between phase 1 and phase 2 of the EMAS cohort, selected hormone parameters from the available phase 1 samples (N=974) from four centers were used in unadjusted binomial logistic regression to determine how well these might predict the morbidity status in phase 2 (**Figure 4**). Unlike the previous analyses, the hormone parameters were first converted to binomial categorical variables, assessing any association of the lowest (INSL3 (<0.7ng/ml), T (<12.1nmol/l), cFT (<236pmol/l), T/LH ratio (<2.12)) or highest (LH (>1.93IU/l)) parameter quartiles of the phase 1 data with morbidity occurrence in phase 2. Interestingly, reduced INSL3, like reduced FT, showed significant Odds Ratios for seven out of nine separate morbidity categories, whereas reduced T, elevated LH, or a reduced T/LH ratio predicted morbidity in only three, five, and four categories, respectively. Only cFT showed any relationship with the index of depression (BDI), and only INSL3 and cFT predicted a relationship with *overall sexual function* (*osf*) and reduced bone mineral density (BMD) (**Figure 4**). Of the five hormone parameters assessed, only increased LH had any predictive value in regard to reduced physical activity and, like INSL3, also showed a significant association with ever having had cancer.

## Discussion

INSL3 is a secreted peptide produced exclusively in the male by the steroidogenic Leydig cells of the testes. Being effectively constitutive, it does not suffer the biological variance attributed to T which may vary considerably across the day or longer within individuals. As a Leydig cell product it depends only on the number and differentiation status of these and hence at a population level may also correlate with T as well as LH, or the T/LH ratio (4, 6), though it is acutely independent of control by the HPG axis. Consequently, circulating INSL3 is a measure of testicular steroidogenic capacity and hence also a sensitive biomarker of primary hypogonadism. Importantly, even though T output may be acutely compensated by increasing LH in cases of compensated hypogonadism (**Figure 1**), INSL3 measurement implies a decreased Leydig cell capacity to produce T, which is not yet reflected by decreased T itself or other indications of hypogonadism (19). This suggests that INSL3 measurement provides additional information about gonadal status compared to T alone.

The present study was carried out to determine whether the ability of INSL3 to act as a biomarker of hypogonadal status also allowed it to predict morbidity, in a similar way to T. Firstly, multiple regression analysis (**Table 1**) showed that while reduced INSL3 does not itself contribute to frailty as a continuous variable (PASE index), it does significantly contribute to calcaneal (heel) bone mass assessed as both calculated bone mineral density (BMD) and bone ultrasound attenuation (BUA). In contrast, this analysis showed that T only contributed to the former parameter, whereas cFT, like INSL3, also contributed to both BMD and BUA (**Supplementary Table S2**), largely because of the involvement in the latter of SHBG (cf. **Table 1**). This apparently independent effect of INSL3 on bone parameters is in agreement with other studies showing that mutations in either the INSL3 gene or that of its receptor, RXFP2, in mice or in humans is accompanied by significant osteopenia or osteoporosis (24), probably due to direct positive effects of the hormone on bone cell metabolism (25).

Reduced T in hypogonadism is also associated with increased symptoms of sexual dysfunction, these being an essential symptomatic in considering any T therapy (26). Whether INSL3 is also predictive of sexual dysfunction is not known and hence correlation analysis was applied. When unadjusted (**Table 2A**), in fact INSL3 (R=0.231) performs better that T (R=0.116), though not as well as cFT (R=0.270), FSH (R=-0.263), or the T/LH ratio (R=0.250) in regard to *overall sexual function* (*osf*). This ability of INSL3 is reduced (R=0.067), however, on adjustment for age (**Table 2B**), indicating that it is largely the mutual association with age together with the low within-individual variance which accounts for the initial good correlation. Similarly, although INSL3 indicates a significant correlation with *change in sexual function* (*csf*) when unadjusted (**Table 2A**), adjustment for age eliminates all parameters from the correlation (**Table 2B**). Interestingly, INSL3 is one of only very few parameters to contribute to the frequency of masturbation index (*m*), suggesting that gonadal status is indeed important in this context. In contrast, the hormones of the HPG axis (T, cFT, LH, FSH, T/LH ratio) indicate no relationship.

In order to assess any direct involvement in sexual function, again multiple regression analysis was carried out (**Table 2C**). This confirmed the very marked negative impact of subject age on *osf*, *csf* and *m*. Focusing on *osf* as the key sexual function parameter with 32.4% of the variance being contributed by the parameters assessed, then INSL3 is seen not to contribute, unlike T, SHBG, LH, smoking, or alcohol. Curiously, INSL3 appears to contribute just significantly to the very small accountable variance for *m*, though to no other sexual parameter, in agreement with the partial correlation analysis (**Table 2B**). Why this should be so is not clear. Substituting cFT for T and SHBG in the analysis has no effect on these results, except that cFT becomes a small contributing parameter also to *csf* (not shown). Taken together, these findings support the view that INSL3 probably relates to sexual function only through its mutual association with age and T or cFT production.

Finally, INSL3 was assessed regarding its predictive ability in the context of more general morbidity, exemplified by the categorical variables listed in **Figure 2**. Using unadjusted univariate binomial logistic regression shows that phase 2 INSL3 performs almost as well as cFT, LH or the T/LH ratio in predicting the occurrence of a range of diverse morbidities. Only the depression index (BDI) is not related. In contrast, T is associated with only 6 of the 9 morbidity categories. However, when multivariate binomial logistic regression is applied (**Figure 3**), which looks at the specific contribution of each parameter to the overall variance from a list of ten potentially involved factors, and hence implying a possible causality, then elevated age and BMI are seen to be the most influential factors, followed by smoking, low T and alcohol intake. Significantly, INSL3 is still seen to be associated with both hypertension and cardiovascular disease, though to no other morbidity. Interestingly, it is increasing INSL3 which is associated with an increased odds for these conditions, for which there is no obvious explanation, especially as it is otherwise hypogonadal status (low T, high LH) as well as high BMI and increased age, which associate with hypertension and cardiovascular disease in the same analysis. This suggests that the effect of INSL3 is not due to its association with the hormones of the HPG axis, but to some other physiological aspect. It is also notable that of all the hormone parameters, only low INSL3 is significantly associated with reduced bone mineral density (**Figure 3**).

The two phases of the EMAS study also allow the testing of true predictive ability, by determining whether parameters measured in phase 1 are associated with morbidity status in phase 2 (**Figure 4**). Here it is notable to see that low INSL3 ranks equivalent to low cFT in predicting 7 out of 9 conditions. In contrast, low T is predictive for only 3 out of 9. LH and the T/LH ratio are intermediate. Comparison with the contemporary association analysis (**Figure 2**) suggests that much of this predictive ability of INSL3 is not only due to it being a biomarker of primary hypogonadal status, but also to its low within-individual biological variance. However, it is important to note that a gap of 4.3 years (average) is not very long considering the chronic nature of many of the reported conditions and their possibly causative impact on inducing hypogonadism (27). Future longitudinal studies should focus on longer time periods to determine whether INSL3 measured in younger or middle-aged men, still without extant morbidity, is truly predictive of the later appearance of age-dependent health issues. In that case, early INSL3 measurement would offer an important preventative parameter to assess male health progression.

There is still very little known about the physiological roles of INSL3 in the adult male, and therefore whether reduced INSL3 may not only be a biomarker for testicular hypogonadism, and therefore reduced T-producing capacity, but also have importance in its own right. Preliminary evidence suggests four possible functions for INSL3 in the adult, acting through its specific receptor, RXFP2. Firstly, and strongly supported by the present findings, INSL3 is known to promote bone metabolism, with genetic defects in the receptor RXFP2 in mice and humans both leading to osteoporosis and osteopenia (24), and with clear effects on bone cells in culture (25). Next, recent evidence suggests a role for INSL3 in the liver to reduce fibrosis (28), and in the kidney to promote healthy kidney function (29). Finally, RXFP2 receptors are known to be located within the testes, not only on germ cells, where they are believed to promote spermatogenesis, but also on the Leydig cells themselves (30). The homologous cells in the female are the steroidogenic theca interna cells of ovarian follicles, which also make and respond to INSL3 (31). In the ovary it has been shown that this local INSL3-RXFP2 system is essential for the production of steroid precursors (31). It is tempting to imagine that something similar may be happening in the testes, and that INSL3 is involved in an autocrine fashion in promoting T production. Thus, a reduction in INSL3 synthesis by the Leydig cells might itself lead to a reduced capacity to make T, thereby exacerbating the hypogonadism. A study in mice lends weight to this hypothesis (32). This concept of INSL3 functionality in the adult is important in view of the application of T hormone replacement therapy. If such therapy is extended to a wider group of men than is advocated at present, then the consequent down-regulation of the HPG axis by the resultant negative feedback, and hence possible Leydig cell involution, would also lead to a further reduction in INSL3 secretion and possible negative side-effects in other organs, as described above.

The debate is continuing whether T or cFT is a better diagnostic marker of functional hypogonadism (33, 34). The EMAS data emphasize the role of cFT as a more sensitive marker by reducing the number of false positive findings, especially when total T levels are borderline suppressed. Although the reasons are unclear, the current results indicate a better correlation of INSL3 with cFT rather than with T. Further work is therefore needed to confirm and define the position of INSL3 in the diagnosis of functional hypogonadism, and in predicting the appearance and progression of this condition. Taken together, the results suggest that INSL3 represents a new and important independent parameter with which to identify primary Leydig cell insufficiency and hence primary hypogonadism, and complementing the use of LH and/or T, which may be subject to influences at central/hypothalamic, pituitary, or on feedback systems.

The strengths of this study are the large and comprehensive dataset provided by the EMAS cohort, together with the accuracy of the hormonal parameters measured. The weaknesses are the self-reported nature of some of the morbidity parameters as well as the relatively short longitudinal dimension of only 4.3 years average. A future study would need to extend this longitudinal component and use more objective measures of morbidity.

## Data availability statement

The raw data supporting the conclusions of this article will be made available by the authors on reasonable request.

## Ethics statement

Ethical approval for this retrospective study was obtained in accordance with local institutional requirements in each of the 8 European centers. The patients/participants provided their written informed consent to participate in this study.

## Author contributions

RA-I supervised the INSL3 measurement and its validation, and contributed to conception, analysis, and writing of this study. KH was responsible for INSL3 measurement. KS advised and supported the statistical analysis. IH and FW were responsible for provision and coordination of EMAS samples and data, and contributed to conception and writing of this study. LA, GB, FC, AG, MM, DO’C, TO’N, MP, GR, JS-H, JT, and DV helped with data collection, reviewing, commenting on and approving the final manuscript. RI was responsible for overall conception, analysis, and writing of the study. All authors contributed to the article and approved the submitted version.

## Funding

We are grateful to the European 5th Framework program for initiation and support of the EMAS project and the German Research Council (DFG; grant no. IV 7/13-1) for funding the INSL3 analysis.

## Acknowledgments

We should like to thank all the men of the EMAS cohort who participated in this study, as well as the technical and medical staff within the EMAS consortium who made this study possible.

## Conflict of interest

The authors declare that the research was conducted in the absence of any commercial or financial relationships that could be construed as a potential conflict of interest.

## Publisher’s note

All claims expressed in this article are solely those of the authors and do not necessarily represent those of their affiliated organizations, or those of the publisher, the editors and the reviewers. Any product that may be evaluated in this article, or claim that may be made by its manufacturer, is not guaranteed or endorsed by the publisher.

## Supplementary material

Supplementary Table 1 Descriptive statistics of relevant parameters measured for the available samples for phases 1 and 2 of the EMAS cohort. Data are expressed as means ± SD (number of subjects).

Supplementary Table 2 Multiple regression analysis modelling the PASE (Physical Activity Scale for the Elderly) index, as well as BMD (bone mineral density), SOS (ultrasound speed of sound), and BUA (bone ultrasound attenuation) for heel bone ultrasound assessment as continuous dependent variables, using hormonal, anthropometric, and lifestyle parameters as independent variables, as indicated, including INSL3. std.b is the standardized beta coefficient for the model with its t value and significance (p value). excl. indicates a parameter excluded in the final optimal model. This analysis is essentially similar to that in **Table 1**, except that cFT now replaces T and SHBG as informative parameters.
