# Supplementary material for: The Leydig cell biomarker INSL3 as a predictor of age-related morbidity: Findings from the EMAS cohort
Source: Front Endocrinol (Lausanne). 2022 Nov 8;13:1016107. doi: 10.3389/fendo.2022.1016107 (PMC9679513; doi:10.3389/fendo.2022.1016107)
Supplement: Supplementary Table 1 — Descriptive statistics of relevant parameters measured for the available samples for phases 1 and 2 of the EMAS cohort. Data are expressed as means ± SD (number of subjects). [file DataSheet_1.pdf]

Supplementary Table Legends

Suppl. Table 1
Descriptive statistics of relevant parameters measured for the available samples for phases 1 and 2 of the EMAS cohort. Data are expressed as means ± SD (number of subjects).

Suppl. Table 2
Multiple regression analysis modelling the PASE (Physical Activity Scale for the Elderly) index, as well as BMD (bone mineral density), SOS (ultrasound speed of sound), and BUA (bone ultrasound attenuation) for heel bone ultrasound assessment as continuous dependent variables, using hormonal, anthropometric, and lifestyle parameters as independent variables, as indicated, including INSL3. **std.b** is the standardized beta coefficient for the model with its t value and significance (p value). **excl**. indicates a parameter excluded in the final optimal model. This analysis is essentially similar to that in Table 1, except that cFT now replaces T and SHBG as informative parameters.

Suppl. Table 1

	Phase 1	Phase 2
**Age (years)**	58.5±10.6 (927)	63.0±10.5 (2325)
**INSL3 (ng/ml)**	1.03±0.50 (927)	0.99±0.50 (2283)
**T (nmol/l)**	16.04±5.43 (927)	16.38±6.05 (2325)
**cFT (pmol/l)**	291.6±85.3 (927)	284.2±90.2 (2283)
**SHBG (nmol/l)**		44.44±20.10 (2283)
**LH (IU/l)**	5.92±3.69 (926)	6.29±4.74 (2282)
**FSH (IU/l)**		8.51±9.33 (2242)
**T/LH ratio**	3.40±1.83 (926)	3.49±3.94 (2282)
**BMI (kg/m^2)**	27.81±3.83 (923)	27.85±4.24 (2231)
**WC (cm)**	98.3±10.4 (926)	99.8±11.5 (2253)
**smoking (%)**	19.9 (918)	17.6 (2288)

Suppl. Table 2 – Multiple Regression Analysis – Bone parameters

	**PASE** (10.6%)			**BMD** (3.9%)			**SOS** (0.2%)			**BUA** (4.7%)		
	**std. β**	**t**	***p*-value**	**std. β**	**t**	***p*-value**	**std. β**	**t**	***p*-value**	**std. β**	**t**	***p*-value**
**age**	-0.326	-14.14	<0.001	-0.086	-3.38	<0.001	-0.049	-2.12	0.034	excl.	excl.	excl.
**INSL3**	excl.	excl.	excl.	0.066	2.50	0.012	excl.	excl.	excl.	0.066	2.53	0.011
**cFT**	excl.	excl.	excl.	0.059	2.25	0.024	excl.	excl.	excl.	0.058	2.22	0.026
**LH**	excl.	excl.	excl.	excl.	excl.	excl.	excl.	excl.	excl.	excl.	excl.	excl.
**FSH**	excl.	excl.	excl.	excl.	excl.	excl.	excl.	excl.	excl.	-0.072	-2.99	0.003
**smoking**	-0.100	4.29	<0.001	-0.059	2.52	0.012	excl.	excl.	excl.	-0.088	-3.83	<0.001
**alcohol**	excl.	excl.	excl.	0.049	2.16	0.031	excl.	excl.	excl.	0.045	1.96	0.050
**BMI**	-0.072	-3.16	0.002	0.120	5.13	<0.001	excl.	excl.	excl.	0.145	6.20	<0.001
